# *ALKBH5* gene polymorphisms and risk of neuroblastoma in Chinese children from Jiangsu Province

**DOI:** 10.1002/cai2.103

**Published:** 2023-12-22

**Authors:** Qian Guan, Xinxin Zhang, Jiabin Liu, Chunlei Zhou, Jinhong Zhu, Haiyan Wu, Zhenjian Zhuo, Jing He

**Affiliations:** ^1^ Department of Pediatric Surgery, Guangzhou Institute of Pediatrics, Guangdong Provincial Key Laboratory of Research in Structural Birth Defect Disease, Guangdong Provincial Clinical Research Center for Child Health, Guangzhou Women and Children's Medical Center Guangzhou Medical University Guangzhou Guangdong China; ^2^ Department of Pathology Children's Hospital of Nanjing Medical University Nanjing Jiangsu China; ^3^ Department of Clinical Laboratory, Biobank Harbin Medical University Cancer Hospital Harbin Heilongjiang China; ^4^ Laboratory Animal Center, School of Chemical Biology and Biotechnology Peking University Shenzhen Graduate School Shenzhen Guangdong China

**Keywords:** *ALKBH5*, neuroblastoma, polymorphism, susceptibility

## Abstract

**Background:**

Neuroblastoma is one of the most common extracranial malignant solid tumors in children. AlkB homolog 5 (ALKBH5) is an RNA N6‐methyladenosine (m6A) demethylase that plays a critical role in tumorigenesis and development. We assessed the association between single nucleotide polymorphisms (SNPs) in *ALKBH5* and the risk of neuroblastoma in a case‐control study including 402 patients and 473 non‐cancer controls.

**Methods:**

Genotyping was determined by the TaqMan method. The association between *ALKBH5* polymorphisms (rs1378602 and rs8400) and the risk of neuroblastoma was evaluated using the odds ratio (OR) and 95% confidence interval (CI).

**Results:**

We found no strong association of *ALKBH5* rs1378602 and rs8400 with neuroblastoma risk. Further stratification analysis by age, sex, primary site, and clinical stage showed that the rs1378602 AG/AA genotype was associated with a lower risk of neuroblastoma in males (adjusted OR = 0.58, 95% CI = 0.35–0.97, *p* = 0.036) and children with retroperitoneal neuroblastoma (adjusted OR = 0.58, 95% CI = 0.34–0.98, *p* = 0.040).

**Conclusions:**

*ALKBH5* SNPs do not seem to be associated with neuroblastoma risk. More studies are required to confirm this negative result and reveal the relationship between gene polymorphisms of the m6A modifier *ALKBH5* and neuroblastoma.

## Graphical abstract

A case‐control study was conducted in 402 neuroblastoma patients and 473 controls in Nanjing Children's Hospital. Genotyping was performed by Taqman method. Based on eQTL analysis and multiple logistic regression model, we found that *ALKBH5* gene polymorphisms (rs1378602 and rs8400) were only weakly associated with the risk of neuroblastoma.

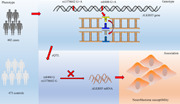

**Abbreviations**
ALKBH5: AlkB homolog 5
CI: confidence interval
GWASs: genome‐wide association studies
HWE: Hardy‐Weinberg equilibrium
m6A: N6‐methyladenosine
OR: odds ratio
SNPs: single nucleotide polymorphisms
UTR: untranslated region

## 1 INTRODUCTION

Neuroblastoma is an extracranial malignant solid tumor originating from primitive neural crest cells in the neuroectoderm [1]. The occurrence rate of neuroblastoma is approximately 1/7000 in the USA and 1/13000 in China. This childhood malignancy accounts for 8%–10% of all pediatric tumor diseases and has a mortality rate of approximately 15% [2, 3]. Neuroblastoma has diverse and complex biological features. Clinically, neuroblastoma is characterized by a relatively hidden primary site, low early diagnosis rate, frequent recurrence rate, and frequent distant metastasis [4, 5]. Characteristics such as age at diagnosis, stage of disease, and nonrandom chromosomal aberrations are used for risk stratification [6]. The therapeutic efficacy of treatment and patient prognosis depend primarily on risk stratification [7, 8]. Despite aggressive multimodal therapy, including radiation therapy, intensive chemotherapy, and surgery, while low‐risk patients have a survival rate of 100%, high‐risk patients have a 5‐year survival rate of less than 50% [9, 10].

While the pathogenesis of neuroblastoma is still unclear, some studies have shown that its carcinogenesis is attributed to interactions between genetic and environmental factors [11, 12]. As neuroblastoma is characterized by genomic instability and chromosomal abnormalities, genetic factors seem to have a more important role in its pathogenesis [13]. Single nucleotide polymorphisms (SNPs) are a major source of genetic and phenotypic variation in humans. Genome‐wide association studies (GWASs) have identified many neuroblastoma susceptibility alleles including those for genes *NEFL* [14], *LIN28B* [15], *BARD1* [16], and *LMO1* [17, 18] and neuroblastoma susceptibility isogenic polymorphisms. Because the SNPs identified by GWASs have only moderate risk effects, exploring more new genetic variants is necessary.

One of the most universal and frequent modifications of RNAs in eukaryotes is N6‐methyladenosine (m6A), which is involved in RNA splicing, RNA folding, RNA stabilization, mRNA translation, and RNA‐protein interactions [19, 20, 21, 22]. AlkB homolog 5 (ALKBH5) is a m6A demethylase, or “eraser” protein, that affects mRNA metabolism, export, and processing factor assembly in the nucleolus by removing m6A modifications. m6A modification of RNA is then recognized and transmitted by “reader” proteins [23, 24]. ALKBH5 has been tightly linked to the occurrence and development of a variety of cancers and acts as a cancer‐promoting or cancer‐suppressing factor [25, 26]. However, the impact of gene polymorphisms in *ALKBH5* on neuroblastoma susceptibility has not been reported thus far [27]. To elucidate the association between *ALKBH5* and neuroblastoma risk, we analyzed potentially functional *ALKBH5* SNPs in this case‐control study.

## 2 METHODS

### 2.1 Ethics approval

Before commencement of the study, we obtained approval from the appropriate institutional review board of Children's Hospital of Nanjing Medical University (Approval No: 202112141‐1). Informed written consent was obtained from all participants or their legal guardians.

### 2.2 Study population

A total of 402 neuroblastoma cases and 473 healthy controls were included in the study (Table S1). The subjects were described in detail in previous studies [28, 29]. In brief, cases recruited from Nanjing Children's Hospital were diagnosed with primary neuroblastoma by histopathology; there was no history of malignant tumors in other organs. The selected children did not receive chemotherapy before blood collection. Controls were healthy subjects who were matched to the case group for age, sex, and city of residence and had no history of neuroblastoma.

### 2.3 SNP selection and genotyping

We screened and identified two potentially functional SNPs (rs1378602 G > A and rs8400 G > A) in the *ALKBH5* gene using the NCBI SNP database (http://www.ncbi.nlm.nih.gov/projects/SNP) and SNPinfo (http://snpinfo.niehs.nih.gov/snpfunc.htm). The specific selection principles were described previously [30, 31, 32]. Both polymorphisms were located in the 3′ untranslated region (UTR), and these two polymorphisms can affect miRNA binding site activity. There was no significant linkage disequilibrium between the selected polymorphisms (R^2^ = 0.113). All samples were genotyped using the TaqMan probe. To ensure genotyping accuracy, we randomly selected 10% of the samples for regenotyping, and the results were 100% consistent with the previous results.

### 2.4 Statistical analysis

The goodness‐of‐fit χ^2^ test was used to test whether the chosen SNPs in the control group deviated from Hardy‐Weinberg equilibrium (HWE). A two‐sided χ^2^ test was adopted to evaluate demographic variables and gene frequencies in the subjects. We evaluated the association between the SNPs of *ALKBH5* and susceptibility to neuroblastoma by calculating odds ratios (ORs) and corresponding 95% confidence intervals (CIs) using unconditional univariate logistic regression analysis. We used the SAS statistics package (version 9.4; SAS Institute) to perform all statistical analyses. A *p* value < 0.05 was considered statistically significant.

## 3 RESULTS

### 3.1 Association of *ALKBH5* gene polymorphisms with neuroblastoma risk

The demographic characteristics of the 402 neuroblastoma patients and 473 controls recruited in Jiangsu are shown in Table S1. Genotyping was successful for all of the included subjects. The results showed no significant difference in the genotype frequency distribution of the rs1378602 G > A and rs8400 G > A polymorphisms between the cases and controls (Table 1). Thus, the two SNPs were not associated with risk of neuroblastoma. We further investigated the effect of both *ALKBH5* risk genotypes on neuroblastoma risk. The results showed that individuals with risk genotypes did not have an increased risk of neuroblastoma compared with individuals without risk genotypes.

**Table 1:** Logistic regression analysis of the associations between *ALKBH5* gene polymorphisms and neuroblastoma susceptibility in children from Jiangsu Province.

Genotype	Cases (*N* = 402)	Controls (*N* = 473)	*p* a	Crude OR (95% CI)	*p*	Adjusted OR (95% CI)^b^	*p* b
rs1378602 (HWE = 0.731)							
GG	339 (84.33)	383 (80.97)		1.00		1.00	
AG	55 (13.68)	86 (18.18)		0.72 (0.50–1.05)	0.084	0.72 (0.50–1.05)	0.084
AA	8 (1.99)	4 (0.85)		2.26 (0.67–7.57)	0.186	2.26 (0.67–7.58)	0.186
Additive			0.443	0.88 (0.65–1.21)	0.443	0.88 (0.65–1.21)	0.443
Dominant	63 (15.67)	90 (19.03)	0.183	0.79 (0.55–1.13)	0.193	0.79 (0.56–1.13)	0.193
Recessive	394 (98.01)	469 (99.15)	0.147	2.38 (0.71–7.96)	0.159	2.38 (0.71–7.97)	0.159
rs8400 (HWE = 0.283)							
GG	127 (31.59)	152 (32.14)		1.00		1.00	
AG	190 (47.26)	222 (46.93)		1.02 (0.76–1.39)	0.877	1.03 (0.76–1.39)	0.876
AA	85 (21.14)	99 (20.93)		1.03 (0.71–1.49)	0.886	1.03 (0.71–1.49)	0.888
Additive			0.877	1.02 (0.84–1.22)	0.877	1.02 (0.84–1.22)	0.877
Dominant	275 (68.41)	321 (67.86)	0.864	1.03 (0.77–1.36)	0.864	1.03 (0.77–1.37)	0.863
Recessive	317 (78.86)	374 (79.07)	0.938	1.01 (0.73–1.40)	0.938	1.01 (0.73–1.40)	0.940
Risk genotypes^c^							
0	127 (31.59)	152 (32.14)		1.00		1.00	
1	267 (66.42)	317 (67.02)		1.01 (0.76–1.34)	0.956	1.01 (0.76–1.34)	0.955
2	8 (1.99)	4 (0.85)		2.39 (0.70–8.13)	0.162	2.39 (0.70–8.14)	0.162
1–2	275 (68.41)	321 (67.86)	0.864	1.03 (0.77–1.36)	0.864	1.03 (0.77–1.37)	0.863

Abbreviations: CI, confidence interval; HWE, Hardy‐Weinberg equilibrium; OR, odds ratio.

^a^ χ^*2*^ test for genotype distributions between neuroblastoma cases and cancer‐free controls.

^b^ Adjusted for age and sex.

^c^ Risk genotypes were carriers with rs1378602 AA (1) and rs8400 AG/AA (1) genotypes when the rs1378602 GG/AG (0) and rs8400 GG (0) genotypes were used as reference.

### 3.2 Stratification analysis

We performed stratification analysis to assess the effects of age, sex, tumor location, and clinical stage on the relationship between selected gene polymorphisms and neuroblastoma susceptibility (Table 2). Compared with the GG genotype, the rs1378602 AG/AA genotypes were related with increased risk of neuroblastoma in the following subgroups: males (adjusted OR = 0.58, 95% CI = 0.35–0.97, *p* = 0.036) and patients with tumors originating from the retroperitoneum (adjusted OR = 0.58, 95% CI = 0.34–0.98, *p* = 0.040).

**Table 2:** Stratification analysis for the association between *ALKBH5* gene polymorphisms and neuroblastoma susceptibility.

	rs1378602 (cases/controls)		Adjusted OR^a^ (95% CI)	*p* a	rs8400 (cases/controls)		Adjusted OR^a^ (95% CI)	*p* a	Risk genotypes (cases/controls)		Adjusted OR^a^ (95% CI)	*p* a
Variables	GG	AG/AA			GG	AG/AA			0	1–2		
Age, month												
≤18	117/114	22/25	0.86 (0.46–1.61)	0.638	44/51	95/88	1.25 (0.76–2.06)	0.373	44/51	95/88	1.25 (0.76–2.06)	0.373
>18	222/269	41/65	0.76 (0.50–1.17)	0.220	83/101	180/233	0.94 (0.66–1.33)	0.726	83/101	180/233	0.94 (0.66–1.33)	0.726
Sex												
Female	155/185	36/40	1.07 (0.65–1.77)	0.778	55/70	136/155	1.12 (0.73–1.70)	0.610	55/70	136/155	1.12 (0.73–1.70)	0.610
Male	184/198	27/50	**0.58 (0.35–0.97)**	**0.036**	72/82	139/166	0.95 (0.65–1.41)	0.807	72/82	139/166	0.95 (0.65–1.41)	0.807
Sites of origin												
Adrenal gland	71/383	22/90	1.32 (0.77–2.24)	0.313	27/152	66/321	1.16 (0.71–1.88)	0.561	27/152	66/321	1.16 (0.71–1.88)	0.561
Retroperitoneal	147/383	20/90	**0.58 (0.34–0.98)**	**0.040**	59/152	108/321	0.87 (0.60–1.26)	0.451	59/152	108/321	0.87 (0.60–1.26)	0.451
Mediastinum	100/383	20/90	0.85 (0.50–1.45)	0.551	35/152	85/321	1.15 (0.74–1.79)	0.525	35/152	85/321	1.15 (0.74–1.79)	0.525
Others	17/383	1/90	0.25 (0.03–1.93)	0.185	4/152	14/321	1.67 (0.54–5.17)	0.372	4/152	14/321	1.67 (0.54–5.17)	0.372
Clinical stages												
I + II + 4 s	142/383	31/90	0.92 (0.59–1.45)	0.719	57/152	116/321	0.95 (0.65–1.38)	0.777	57/152	116/321	0.95 (0.65–1.38)	0.777
III + IV	137/383	26/90	0.81 (0.50–1.30)	0.375	49/152	114/321	1.10 (0.75–1.62)	0.640	49/152	114/321	1.10 (0.75–1.62)	0.640

*Note*: The results in bold indicate *p* values less than 0.05 or 95% CI excluded 1.00.

Abbreviations: CI, confidence interval; OR, odds ratio.

^a^ Adjusted for age and sex, omitting the correspondence factor.

## 4 DISCUSSION

m6A is one of the most common modifications of RNAs in eukaryotes; these modifications are regulated by writers and erasers and recognized by readers [33]. The process of m6A modification is dynamic and reversible and mainly depends on m6A demethylases, which function as erasers [34, 35]. The known demethylases are FTO and ALKBH5 [36]. ALKBH5 regulates mRNA export, RNA metabolism, and assembly of mRNA processing factors by reducing RNA m6A levels in nuclear spots [37]. ALKBH5 inactivation leads to dysregulation of RNA, resulting in abnormal splicing and accumulation of shorter transcripts [38]. ALKBH5 also plays a vital antiviral role. DDX46 demethylates antiviral transcripts by recruiting ALKBH5 and modifying m6A in antiviral transcripts; this results in the retention of antiviral transcripts in the nucleus and prevention of their translation, with a negative role in regulating the inherent antiviral response [39].

To date, there are limited reports on the relationship between SNPs in the *ALKBH5* gene and disease risk. In recent years, with the increase of m6A modification research, *ALKBH5* SNPs were found to be associated with disease susceptibility. Du et al. [40] found that *ALKBH5* SNP rs12936694 may confer susceptibility to major depressive disorder in the Chinese Han population. In a rheumatoid arthritis study, 21 SNPs of *ALKBH5* significantly correlated with risk [41]. Hua et al. [30] assessed the relationship between rs1378602 and rs8400 of the *ALKBH5* gene and Wilms tumor susceptibility in 414 patients with nephroblastoma and 1199 healthy controls in a multicenter case‐control study. The results indicated only a weak connection between these two SNPs and Wilms tumor risk. Some studies have also reported that *ALKBH5* rs2029399, rs298981, rs115267066, rs167246, and rs441216 are not associated with the risk of colorectal cancer [42]. Zhuo et al. [43] investigated genetic variants of *METTL14*, which encodes a m6A methyltransferase, and found that rs62328061 A > G and rs298982 G > A reduce the risk of neuroblastoma while rs4834698 T > C and rs9884978 G > A increase its risk. These studies indicate that ALKBH5 may be related to neuroblastoma.

In this study, we investigated for the first time whether *ALKBH5* SNPs affect the risk of neuroblastoma in Chinese children. Our results found no association between two SNPs (rs8400 and rs1378602) and neuroblastoma risk. There was no risk correlation in joint analysis. Notably, stratified analysis showed that compared with the GG genotype, the AG/AA genotypes were related to a lower risk in males and children with neuroblastoma of retroperitoneal tumor origin. However, the small sample size in the hierarchical analysis may also play a conflicting role. However, one study with 967 cases and 1813 controls from nine centers recently published by our group showed that a variant of *ALKBH5* rs8400 was associated with increased neuroblastoma risk. No statistically significant association was found for rs1378602. Further functional research revealed that the rs8400 variant affects the binding of miR‐186 to the 3′ UTR of *ALKBH5*, changes the expression of *ALKBH5*, and ultimately modifies neuroblastoma susceptibility [44]. These two seemingly contradictory results may be attributed to the differences of sample size and the diversity of the sample source. To further understand the pathogenesis of neuroblastoma and improve prognosis, exploration of more gene polymorphisms that may mediate the risk of neuroblastoma is required.

The main advantage of this study is its innovation. However, this study also has some limitations. First, although the single‐source population weakens the influence of confounding factors and enhances the reliability of the research conclusions, universality is also reduced. Second, in stratified analysis, the sample size was small, and the statistical strength was far from sufficient. Third, because of the retrospective nature of this study, only genetic information for the *ALKBH5* gene was considered, and the influence of environmental factors and clinical features of the cases, such as tumor size, *MYCN* amplification, and metastatic site, were not taken into account. Finally, only two *ALKBH5* SNPs were analyzed. To further understand the pathogenesis of neuroblastoma, exploration of more gene polymorphisms that may modify neuroblastoma risk is required.

## 5 CONCLUSIONS

In conclusion, we explored the relationship between *ALKBH5* gene polymorphisms and susceptibility to neuroblastoma in China. The results show that *ALKBH5* gene polymorphisms may have a weak effect on risk of neuroblastoma. More large‐scale research is needed to verify this result.

## AUTHOR CONTRIBUTIONS

**Qian Guan**: Investigation (equal); Writing—original draft (equal). **Xinxin Zhang**: Conceptualization (equal); Writing—original draft (equal). **Jiabin Liu**: Investigation (equal). **Chunlei Zhou**: Methodology (equal); Writing—review & editing (equal). **Jinhong Zhu**: Project administration (equal); Supervision (equal). **Haiyan Wu**: Formal analysis (equal). **Zhenjian Zhuo**: Funding acquisition (equal); Investigation (equal); Writing—review & editing (equal). **Jing He**: Visualization (equal); Writing—original draft (equal); Writing—review & editing (equal).

## CONFLICT OF INTEREST STATEMENT

Professor Zhenjian Zhuo is the member of the *Cancer Innovation* Editorial Board. To minimize bias, he was excluded from all editorial decision‐making related to the acceptance of this article for publication. The remaining authors declare no conflict of interest.

## ETHICS STATEMENT

The study protocol was approved by the institutional review board of Children's Hospital of Nanjing Medical University (Approval No: 202112141‐1), and it was compliant with the Helsinki Declaration of 1975, as revised in 2008.

## INFORMED CONSENT

All patients or their legal guardians provided written informed consent at the time of entering this study.

## Supporting information

Supporting information.

## Data Availability

Data available on request from the authors.
